# Heterogeneous groups cooperate in public good problems despite normative disagreements about individual contribution levels

**DOI:** 10.1038/s41598-020-73314-7

**Published:** 2020-10-07

**Authors:** Kasper Otten, Vincent Buskens, Wojtek Przepiorka, Naomi Ellemers

**Affiliations:** 1 Department of Sociology, Utrecht University, Padualaan 14, 3584 CH Utrecht, The Netherlands; 2 Department of Psychology, Utrecht University, Utrecht, The Netherlands

**Keywords:** Human behaviour, Psychology and behaviour

## Abstract

Norms can promote human cooperation to provide public goods. Yet, the potential of norms to promote cooperation may be limited to homogeneous groups in which all members benefit equally from the public good. Individual heterogeneity in the benefits of public good provision is commonly conjectured to bring about normative disagreements that harm cooperation. However, the role of these normative disagreements remains unclear because they are rarely directly measured or manipulated. In a laboratory experiment, we first measure participants’ views on the appropriate way to contribute to a public good with heterogeneous returns. We then use this information to sort people into groups that either agree or disagree on these views, thereby manipulating group-level disagreement on normative views. Participants subsequently make several incentivized contribution decisions in a public goods game with peer punishment. We find that although there are considerable disagreements about individual contribution levels in heterogeneous groups, these disagreements do not impede cooperation. While cooperation is maintained because low contributors are punished, participants do not use punishment to impose their normative views on others. The contribution levels at which groups cooperate strongly relate to the average normative views of these groups.

## Introduction

Norms indicate social standards for individual behavior, and are considered to be one of the main factors sustaining cooperation among humans^1–7^. Cooperative goals often require people to bear an individual cost to benefit the group as a whole. This can create a social dilemma where each member is individually best off by free-riding on contributions of others, while the group as a whole is best off if everybody contributes their share. Prosocial contribution norms can solve this social dilemma by prescribing sufficient contributions for the public good. Experiments using linear public goods games (PGGs) show that groups often sustain high contribution levels if they have the opportunity to enforce prosocial contribution norms through peer punishment, whereas they largely fail to do so without options for norm enforcement^8,9^.

However, research suggests that the potential of norms to promote cooperation for public good provision may be limited to homogeneous groups in which individuals can contribute similar amounts and benefit equally from each other’s contributions. Heterogeneous groups in which individuals differ in how much they can contribute to and benefit from the public good often achieve lower levels of cooperation, even if there is an opportunity to enforce contribution norms^10–13^. These lower levels of cooperation are commonly attributed to normative disagreement within heterogeneous groups^12–15^. While homogeneous groups largely agree upon a single appropriate level of contributions, heterogeneity within groups brings about a plurality of different and conflicting views about how much group members should contribute^11^. This normative disagreement has been conjectured to harm cooperation. If group members are dissatisfied with the contributions of others, they may react with lower contributions themselves, leading to outcomes that are worse for everyone^16^. Here we study experimentally whether normative disagreement harms cooperation in heterogeneous groups.

In everyday life, individual heterogeneity is the rule rather than the exception^17^. The importance of incorporating heterogeneity in experiments is increasingly recognized^18–22^. One ubiquitous type of heterogeneity concerns the returns from the public good. For example, the costs of public facilities such as dams and parks are shared by all taxpayers, even if these often provide different benefits to individuals depending on their distance to or frequency of enjoying the facility. In workplaces, employees may differ in their returns from contributing to teamwork^23^, such as researchers benefitting differently from joint publications at different career stages. Countries have different interests and costs in jointly addressing global problems such as climate change^24^ or the refugee crisis^25^.

In all of these examples, there are at least two common and conflicting views about how people ought to behave^11,12,22^. One view is that all actors should contribute equally to the public good, which implies that those obtaining higher returns from the public good also end up earning more. The other view is that the actors with higher returns from the public good contribute more than the others, such that earnings are equalized. For example, some may argue that citizens living in areas with higher risks of flooding should contribute more to the construction of dams, whereas others may argue that all people should contribute equally regardless of where they live. Thus, there is a potential for normative disagreement (sometimes referred to as normative conflict) in heterogeneous groups between the views of equal-contributions and equal-earnings.

Lab experiments can manipulate the level of normative disagreement in a controlled decision environment, which helps to isolate its impact on cooperation from potential confounders that exist in real-life contexts^26^. The PGG is a classic laboratory paradigm for studying cooperation problems in groups. In previous experimental research on the PGG, the existence of normative disagreement and its influence on cooperation were mainly inferred indirectly from the way people contributed to the public good and sanctioned each other^11–15,27–29^. Normative views were rarely measured in the PGG (see for exceptions^11,30–34^). This means that, to date, we have limited information on what people actually think constitutes appropriate contribution behavior, or how this might influence the choices they make. What is more, to our knowledge, group disagreement on these normative views has not been manipulated experimentally.

The role of normative disagreement in explaining (the lack of) cooperation in heterogeneous groups therefore remains ambiguous, and alternative explanations cannot be ruled out. For example, heterogeneity in groups may also be related to confusion about the cooperation problem, difficulty with coordinating behavior among group members, or the existence of inefficient norms, all of which could negatively affect cooperation^10,33,35^. Hence, without explicitly measuring and manipulating normative disagreement, our understanding of its causal influence on cooperation remains limited. This research makes two main contributions. First, we explicitly measure normative views in the PGG and analyze their content. Second, we manipulate whether groups agree or disagree on these normative views and examine the causal influence of this (dis)agreement on cooperation in terms of contributions to the public good.

**Figure 1:**
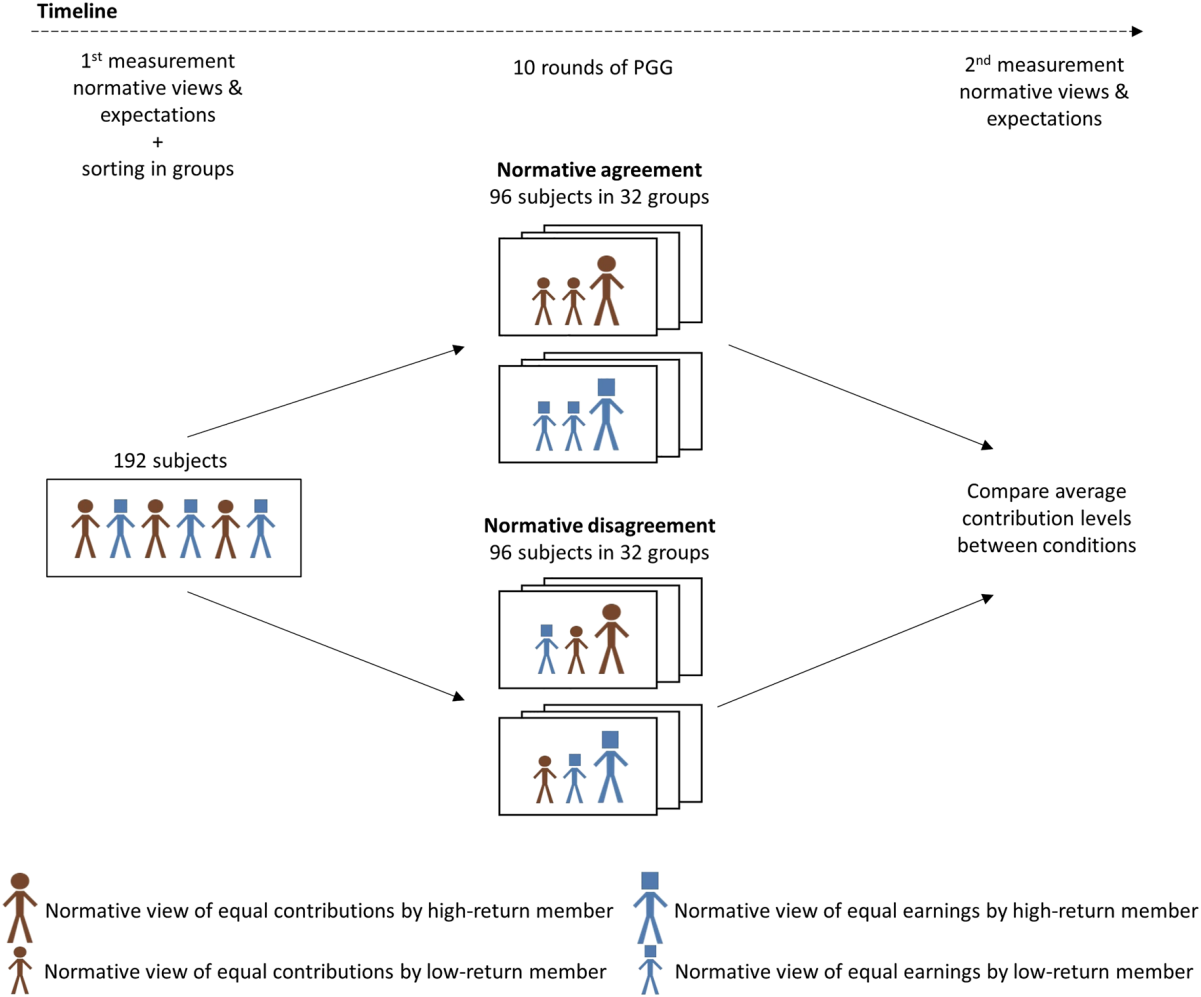
Experimental setup. *Note* We ran 8 sessions with 24 participants each, leading to a total sample of 192 participants. Randomization took place at the session-level. Within each session, participants reported their normative views before and after playing the game and were sorted on a continuum from equal-contributions to equal-earnings. This is denoted by the red (circle-headed) and blue (square-headed) figures supporting equal-contributions and equal-earnings respectively. The sorting differs by condition. In the condition for normative agreement, participants were sorted with similar others. In the condition for normative disagreement, participants were sorted such that one participant per group disagrees with the two other group members (see Methods). One member per group has a higher return from the public good than the other two, as is illustrated here by differences in body size.

An overview of our experiment is shown in Fig. 1. Participants (*N* = 192) play the PGG in fixed groups of three for 10 rounds. In each round, participants obtain 20 monetary units (MU) and choose how many of these MUs to contribute to a group project and how many to keep for themselves. The contributions of all group members are added up, multiplied by a factor larger than 1, and then distributed among the group members based on individual returns. While the multiplication factor is larger than 1, the individual return of each member is smaller than 1. This constitutes a social dilemma because each member individually is best off by not contributing (retaining their own MUs), while the group as a whole is best off if all members fully contribute. Participants can punish individuals whose contribution decisions they do not approve. They can do so by deducting MUs from group members after each contribution decision^9,11^. As mentioned, normative disagreement can ensue when individuals differ in the benefits they obtain from cooperating. To introduce this possibility, we assign heterogeneous individual returns from the public good. Per group, one member receives a fifty percent higher return than the two other members (0.75 vs 0.50). A prior study suggests that with this level of heterogeneity, there is considerable variation among people in whether they support the rule of equal-contributions or equal-earnings^11^.

Before communicating which participants obtain the low and high returns, we ask participants to report their normative views on the appropriate contributions that the high-return and the low-return members should make. We also ask them to report their normative expectations, i.e., what they expect others to consider appropriate contributions. Participants’ own normative views are used to position them on the spectrum of equal-contributions to equal-earnings. Supporters of equal-contributions would answer that both types of players should contribute equally to the public good, whereas supporters of equal-earnings would answer that high-return types should contribute twice as much as low-return types. Participants who support a balance between both rules would answer that high-return types should contribute more than low-return types, but not twice as much. It turns out that almost all of our participants fall within one of these three categories and are rather evenly distributed across these three categories.

Group-level normative disagreement is manipulated by sorting participants into groups with either similar or dissimilar views (two conditions) on the spectrum of equal-contributions to equal-earnings. That is, in the normative agreement condition, we sort participants from the same side of the spectrum together, whereas in the normative disagreement condition we sort participants from different sides of the spectrum together (see Fig. 1, and for more details Methods). To keep our design comparable to related research^10–13^, we neither inform participants about the normative views of their group members, nor about the method of group formation (see Methods). In both conditions, participants play the PGG within their group for 10 rounds. As we will show, participants’ normative views remain largely stable over the 10 rounds. This implies that the difference in normative disagreement between conditions remains largely stable over time as well. By comparing the average contribution levels between the two conditions, we can assess to what extent normative disagreement negatively influences cooperation in terms of contributions to the public good.

The prevailing theoretical prediction is that normative disagreement has a negative effect on public good provision^10–15^. Many people are conditional cooperators, who contribute only if others are also contributing their share^36,37^. If people contribute according to different normative views while observing others’ contributions, they can discover that their own view is not adhered to by others. The expected consequence is that conditional cooperators who think others are not contributing enough will reduce their own contribution, causing a downward trend in contribution levels. Research on PGGs without punishment finds that contributions decline significantly already when one actor contributes less than others in groups of three^38^, four^39^, and six^40^.

While peer-punishment is known to facilitate cooperation in contexts without normative disagreements, it is not expected to help much in contexts with normative disagreements, and can make matters even worse^14,41^. Previous research shows that, without punishment, contributions steadily decline towards free-riding in all groups, regardless of differences in norms and treatments. However, clear differences in cooperation occur when punishment is possible^11^. Actors who receive punishment while behaving according to their own normative view may deem the punishment unjustified, and retaliate by further reducing their contributions or by counter-punishment^42^. We therefore hypothesize that normative disagreements have a negative influence on public good provision in contexts with punishment possibilities. To test this hypothesis, we focus on public good games with punishment possibilities and do not consider contexts without punishment possibilities.

We start the results section with an overview of the type and distribution of normative views and expectations among our participants. Moreover, we examine the temporal stability of participants’ normative views. Then, we statistically test our hypothesis. Our paper concludes with a brief exploratory analysis and a general discussion of our findings.

## Results

### Describing normative views

To elicit normative views, we showed the participants a hypothetical group of three members, two of which obtain a low-return and one of which obtains a high-return, the exact same composition of returns as used in the actual contribution rounds of the experiment. We subsequently asked: “According to you, what is the appropriate amount that each member should contribute to the group account”. The participants could then indicate a contribution for each of the three members between 0 and 20 (see also Figure S1 in the supplementary material). Because there is no right or wrong normative view, we did not incentivize these decisions. We subsequently elicit participants’ normative expectations by letting them guess what their two other group members’ answers were. The elicitation of normative expectations is incentivized in line with prior studies^43^ to motivate participants to seriously put themselves in the shoes of the other participants (see Methods).

**Figure 2:**
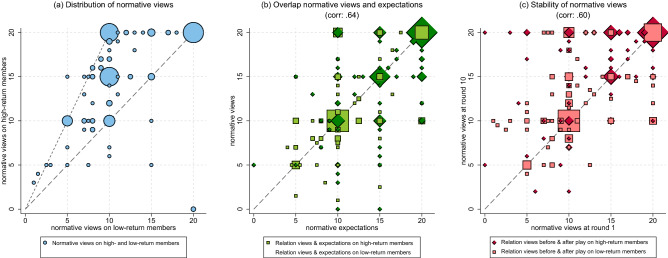
(**a**–**c**) Normative views and expectations: distribution, overlap, and temporal stability. *Note* Number of observations in panel (**a**) is 192, and 384 in panels (**b**, **c**). Marker size is weighted by the number of observations. The marker size in the legends display the size for single participants. Normative views on contributions of high- and low-return members are separated by axis in panel (**a**), and by marker symbol in panel (**b**,**c**) (diamond for views on high-return members, squares for views on low-return members). Although participants were asked to provide their normative view and expectation on the appropriate contribution for each of the two low-return members separately, more than 90% provided the same answer for both low-return members. We therefore average the answers on the two low-return members.

In Fig. 2a, we plot participants’ normative views regarding the appropriate contribution of group members with a low return rate (*x*-axis) against these participants’ normative views regarding the appropriate contribution of group members with a high return rate (*y*-axis). Figure 2a shows that there is considerable heterogeneity in normative views between participants. Yet, as anticipated, virtually all observations fall within the range between equal contributions (the *y* = *x* line) and equal earnings (the *y* = 2*x* line). Three normative views are especially prevalent: (1) equal and full contributions (i.e., collective efficiency), achieved by all group members contributing their full endowment; (2) equal earnings (i.e., equality), achieved by the high-return members contributing their full endowment and the low-return members contributing half of it, and (3) a mix between equal-contributions and equal-earnings, achieved by the high-return members contributing three-quarters of their endowment, 50% more than the low-return members. Each of these normative rules has about the same number of advocates among our participants (about 20% each). We thus find that participants’ normative views map well along the dimensions of equal-contributions and equal-earnings, and that there is substantial heterogeneity among participants on these views.

To examine whether participants expect others to hold the same normative views as themselves, we plot participants’ normative expectations (*x*-axis) against their normative views (*y*-axis) in Fig. 2b. Observations on the diagonal represent participants for which normative views and expectations fully overlap. As can be seen, most participants are on the diagonal; they expect others to hold the same normative views as they do. On average, the correlation between views and expectations is 0.64 (*p* < 0.001). The extent of overlap between normative views and expectations is largely the same for answers on high- and low-return members. In both cases, about 60% of participants expect others to hold exactly the same views as they do, 20% support higher contributions than they expect others to support, and 20% support lower contributions than they expect others to support.

We elicited participants’ normative views at the start and at the end of the ten rounds of the PGG. This allows us to assess the temporal stability of participants’ normative views. In Fig. 2c, we plot participants’ initial normative views (*x*-axis) against their normative views at the end of the ten PGG rounds (*y*-axis). Most observations lie on the diagonal, representing participants with stable normative views. The average correlation between views before and after the ten PGG rounds is 0.60 (*p* < 0.001). Participants whose view matched neither the equal-contributions nor the equal-earnings rule from the start are considerably more likely to change it. Out of the 101 participants who matched one of the two rules at first measurement, only 33 changed them (33%), whereas out of the 91 participants who did not match one of the two rules, 74 changed them (81%) (Mann–Whitney ranksum test, *p* =  < 0.001). About half of the participants who initially did not uphold one of the two focal normative views and then changed their views, switch to one of the two focal rules (34 out of 74 participants). Thus, the two normative rules of equal-contributions and equal-earnings have a substantial number of supporters from the start, and these supporters are unlikely to change their views over time. Participants whose views do not match one of the two focal rules from the start are more likely to change them, and if they do, they often end up supporting one of the two focal rules.

We find little support for self-serving normative views (high-return participants supporting the equal contributions view and low-return participants the equal earnings view because that is in their best interest). Table 1 shows that both high-return and low-return participants report roughly the same normative views, both before and after the ten PGG rounds. We also find no indication that participants are more likely to change their view if it receives minority instead of majority support. Both the absolute and relative change in normative views between the ten PGG rounds are similar for participants holding a minority and majority view (see supplementary material, Table S1). In sum, our descriptive findings suggest that participants hold well-defined normative views about contributions to the public good under return heterogeneity, there is substantial between-participant variation in these views, these normative views largely overlap with normative expectations, and they are mostly stable over time.

**Table 1:** Descriptive statistics on contributions, punishments, and normative views.

	Average	Return of participant		Mann–Whitney test of difference by return-type	
		Low	High	*z*-statistic	*p-*value
Contribution	13.87 (5.72)	12.93 (5.67)	15.74 (5.36)	− 10.78	< .001
Punishment assigned	0.72 (1.87)	0.78 (2.03)	0.58 (1.49)	1.67	.09
Punishment received	0.72 (1.87)	0.61 (1.60)	0.92 (2.31)	− 2.32	.02
**Normative views before game**					
On high-return members	15.76 (4.77)	15.66 (5.04)	15.97 (4.20)	− 0.07	.94
On low-return members	11.92 (4.89)	11.95 (5.05)	11.85 (4.60)	0.47	.64
**Normative views after game**					
On high-return members	17.40 (4.10)	17.75 (3.80)	16.70 (4.60)	1.41	.16
On low-return members	13.08 (4.82)	12.96 (4.80)	13.31 (4.88)	− 0.62	.54

There are two low-return members and one high-return member per group. Standard deviations are in parentheses.

### Normative disagreement and public good provision

The heterogeneity in normative views allowed for a successful manipulation of disagreement at the group-level. To assess how our sorting procedure affected group-level disagreement, we rank participants within each group based on how much more they think high-return members should contribute than low-return members. Group-level disagreement is measured by comparing this difference between the highest-ranked and lowest-ranked participant of each group. In the disagreement condition, the difference supported by the highest-ranked participant was on average 7.70 contribution points larger than that of the lowest-ranked participant. In the agreement condition, the difference supported by the highest-ranked participant was on average only 1.23 contribution points higher than that of the lowest-ranked participant (differene between conditions with Mann–Whitney ranksum test, *p* =  < 0.001).

**Figure 3:**
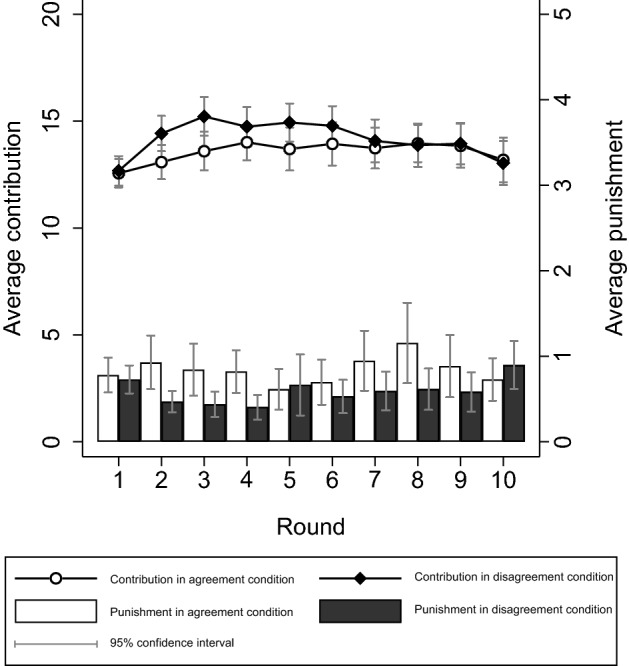
Average contribution and punishment per round and condition.

In Fig. 3, we show the average contribution (left *y*-axis) and punishment level (right *y*-axis) for each condition over the course of the ten PGG rounds. In line with prior research on groups with heterogeneous returns, we find that participants contribute about two-thirds of their endowment on average^11^. This is also in line with the conclusion that public good provision is lower in heterogeneous groups than in homogeneous groups, where the contribution level is typically closer to full contributions when peer-punishment is possible (see also Figure S2 in SI)^8^. However, Fig. 3 suggests that the lower levels of public good provision found in heterogeneous groups are not due to normative disagreement. The contribution levels are similar in both the agreement and disagreement condition, suggesting no support for the hypothesis that normative disagreement harms public good provision.

We use population-averaged regression models, which account for repeated measures obtained from the same participant or group, to statistically test this hypothesis, with the contribution decision as the dependent variable and the experimental condition as the predictive factor. Across six models we vary whether the outcome variable is on the individual-level or group-level, and whether we include all rounds, only the first round (1), or only the last round (10) as observations. Table 2 shows that, regardless of which model is used, we find no significant difference in contribution levels between conditions according to conventional standards (*p* < 0.05, with Bonferroni adjustment for multiple comparisons). Non-parametric (Mann–Whitney) tests lead to the same conclusion (see Table S2 in SI). The hypothesis that normative disagreement harms public good provision is therefore not supported. The between-condition variance in contribution levels is negligible (< 1%), implying that virtually all variation is within conditions. Similarly, Fig. 3 suggests no substantial differences in punishment levels between conditions, which is corroborated by non-parametric (Mann–Whitney) tests (see Table S2 in SI). In both conditions, the average punishment allocated falls mostly between 0.5 and 1 whereas the possible range is from 0 to 10. We find no significant difference in punishment allocation between high-return and low-return participants (see Table 1). When subdividing contribution and punishment levels by the participants’ return rate, we also find no difference between conditions (see supplementary material, Figure S3). In exploratory analyses presented in the supplementary material, we also do not find a significant effect of normative disagreement on contribution levels under alternative conceptualizations of normative disagreement and model specifications (Figure S6 and Tables S3–S5).

**Table 2:** Population-averaged model tests of hypothesis.

	(1)	(2)	(3)	(4)	(5)	(6)
	Ind. all rounds	Ind. start rounds	Ind. end rounds	Group. all rounds	Group. start rounds	Group. end rounds
Treatment (disagreement)	.608	.115	− .156	.608	.115	− .156
	(.672)	(.764)	(.929)	(1.014)	(.831)	(1.274)
Intercept	13.561***	12.563***	13.188***	13.561***	12.563***	13.187***
	(.475)	(.540)	(.657)	(.717)	(.588)	(.901)
*N* observations	1920	192	192	640	64	64
*N* participants	192	192	192			
*N* groups	64	64	64	64	64	64

We use population-averaged regression models to statistically examine the hypothesis that the contribution level is higher in the normative agreement condition than in the normative disagreement condition. The contribution decision is the dependent variable and the experimental condition is the predictive factor. Across six models we vary whether the outcome variable is on the individual-level (models 1–3) or group-level (models 4–6), and whether we include all rounds (models 1 and 4), only the first round (models 2 and 5), or only the end round (models 3 and 6) as observations. Regardless of which model is used, we find no significant difference in contribution levels between conditions according to conventional standards.

*p < 0.05, **p < 0.01, ***p < 0.001 (Bonferroni-adjusted *p/*6, two-tailed tests). Standard errors in parentheses. In model 1 and 4 there are repeated measures on individuals/groups. We take these repeated measures into account with the exchangeable working correlation matrix.

**Figure 4:**
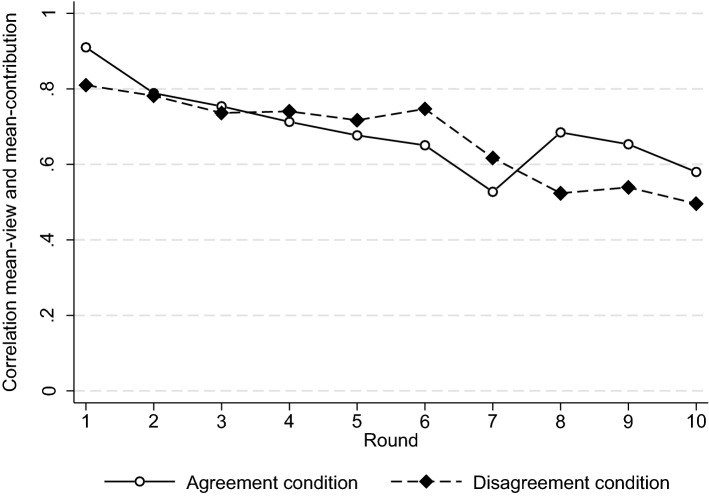
Correlation between group-mean normative views and contributions.

The exploratory analyses show that, instead of normative disagreement, the average normative view of the group strongly relates to the average contribution level that it actually achieves. In Fig. 4, we present the bivariate correlation between group-mean normative views and group-mean contributions per round. We find that in the initial rounds of the game, group-mean normative views almost perfectly predict group-mean contribution levels in both conditions (*r* > 0.8). Although the influence of normative views decreases somewhat over time, it remains substantial throughout the entire 10 rounds (average *r* = 0.66). The normative views participants have before they start interacting with each other thus strongly predict how they behave in the subsequent interactions. In the supplementary material, we show that the association between normative views and contributions is also present when subdividing by return-type, condition, and different normative views (Figure S3–S5). We conclude that the contribution level reached within groups is strongly related to the mean normative views within the group. Low contribution levels are thus not related to the existence of normative disagreement, but rather to the existence of group-mean normative views that support these low levels.

There are multiple reasons for why normative disagreement does not influence public good provision while the group-mean view does. We consider three potential explanations: (1) people quickly adjust their normative views in line with the contributions of their group members, (2) people retain their normative views but reach a contribution level that compromises between the different views, and (3) people do not impose their own normative views on others. Figure 2c suggests that the first explanation is unlikely: most participants hold the same normative views before and after the game (~ 60%). What is more, this likelihood of holding stable normative views does not differ between conditions (Mann–Whitney ranksum test for stability on equal-contributions versus equal-earnings spectrum, *p* = 0.19). Our data also provides little support for the second explanation. If the contribution level is a compromise between group members’ views, the absolute gap between what participants themselves think they should contribute and what they actually contribute should be bigger in the disagreement condition (i.e., where compromise is necessary) than in the agreement condition (i.e., where compromise is not/less necessary). However, this difference is small: the gap is on average 3.19 in the agreement condition and 3.68 in the disagreement condition (Mann–Whitney ranksum test, *p* = 0.04).

**Figure 5:**
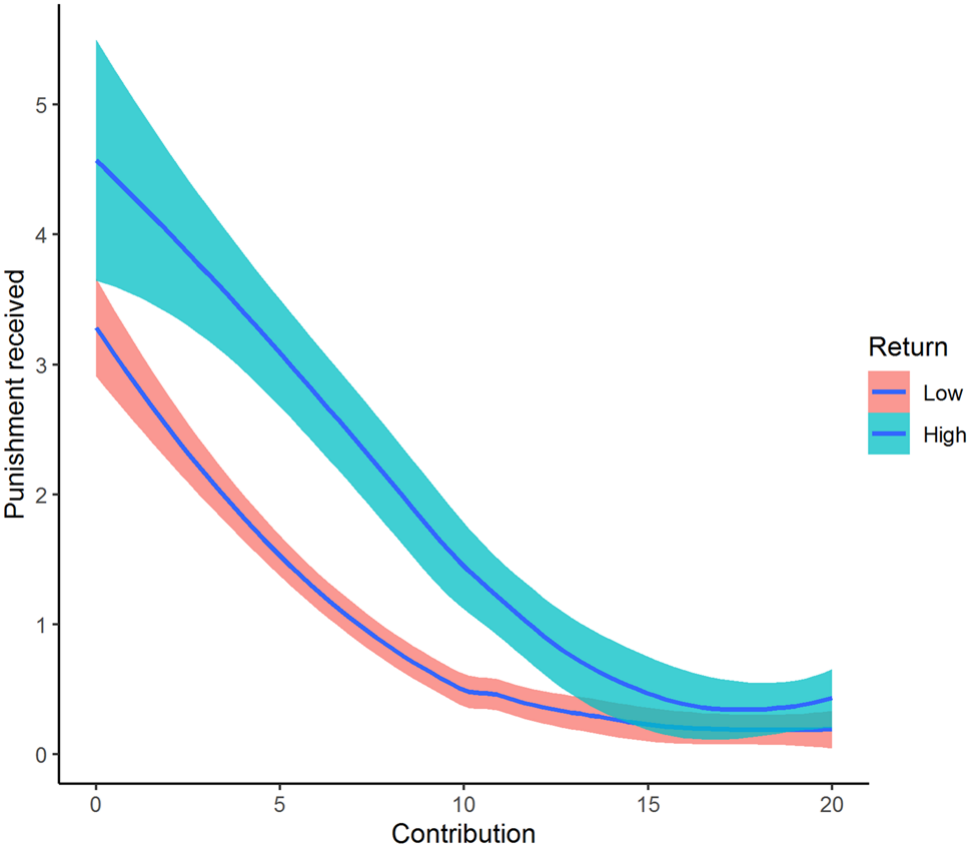
Received punishment as a locally estimated (LOESS) function of contributions with 95% confidence intervals.

To assess the third explanation, that participants do not impose their normative views on others, we turn to punishment behavior. In Fig. 5, we plot the relationship between contributions (*x*-axis) and the received punishment (*y*-axis) for high- and low-return participants. We see that for low-return participants, the received punishment is very low as long as they contribute 10 or more. Recall from Fig. 2a that the most common way to achieve the equal-earnings rule is for low-return members to contribute 10 while high-return members contribute 20, and the most common way to achieve the equal-contribution norm is for all members to contribute 20. Thus, as long as low-return participants do not contribute below a level required to fall between one of the two prosocial contribution norms, they are hardly punished. Yet, when asking about contribution norms, only a minority of participants reports that equal-earnings is the appropriate norm (the majority of participants balance equal-contributions and equal-earnings, see Fig. 2a). This suggests that participants do not impose their own normative views on their group members, as long as the group members’ contributions fall between one of the two prosocial contribution norms. Contributions of 10 by high-return members fit neither of the two prosocial rules (see Fig. 2a) and therefore do lead to punishment. Similarly, free-riding fits neither of the two prosocial rules and is punished regardless of return-type. Post-experiment measurements on normative views of punishment also support the conclusion that participants do not think it appropriate to punish contributions that do not conform to their own normative views, as long as these contributions conform to one of the prosocial views (see SI, Figure S7).

## Discussion

It is commonly conjectured that normative disagreements in heterogeneous groups negatively affect cooperation. To our knowledge, we are the first to directly test this conjecture. In a lab experiment, we measure each participant’s view regarding the appropriate way to contribute to a public good with heterogeneous returns and use this information to manipulate whether members of a group agree or disagree on these normative views. Our results show that participants vary considerably in their normative views, but in a predictable way. Virtually all participants subscribe to a norm of equal-contributions, equal-earnings, or a balance between these two. However, disagreement between these normative views does not negatively affect public good provision. Group composition in terms of normative views does have a strong relationship with the level of public good provision. However, it is the group-mean, rather than the group-disagreement, that matters.

Joint payoffs are maximized when all members contribute fully to the public good, i.e., under the efficient equal-contributions rule. We find, however, that many participants support deviations from the equal-contributions rule to achieve equal-earnings or a balance between the two rules. Notably, in our experiment, equal-earnings could only be achieved by lowering the earnings of high-return members and not by increasing the earnings of the low-return members. Still, many participants supported lowering the joint payoffs to achieve more equalized earnings. Furthermore, participants who do support the equal-contributions rule nevertheless largely refrain from enforcing others to contribute alike. We therefore suggest that there is an alternative reason than normative disagreement for why public goods with heterogeneous returns are underprovided in terms of joint payoffs. Most people support this under-provision to achieve more equalized earnings, and if they do not support it, they refrain from punishing it.

Normative disagreements have been put forth to explain the lack of cooperation in several pressing real-life issues that require parties to invest in a shared public good, such as the climate crisis^44^, the European debt crisis^45^, or the refugee crisis^25^. We used a lab experiment to isolate the effect of normative disagreement from other complexities that arise in such real-life situations. Our results suggest that normative disagreement alone is not sufficient to harm cooperation.

It is possible that normative disagreements would have been harmful under alternative design choices. For example, had we made the differences in returns from the PGG larger or told participants about the (conflicting) normative views of the members they were grouped with, we may have observed a negative effect of disagreement on contribution levels. However, the prior studies that led to the conjecture that normative disagreement causes lower contribution levels used similar levels of heterogeneity and also did not provide participants with information about each other’s normative views^11,12^. We kept our design as close as possible to these experimental designs. Our results thus suggest that under these standard experimental conditions, normative disagreement is not the explanation for lower contribution levels.

Future research can further chart the boundary conditions under which normative disagreements affect cooperation. A promising direction involves manipulating what participants know about others’ views and how they can express their own. We list three suggestions: First, one could manipulate whether participants have direct information on other’s normative views and the level of disagreement. Norm disagreements may become more salient when participants have this information, leading to potentially more harmful effects, e.g., when disagreement triggers attempts to convince other group members that one’s own views are superior. Second, on top of revealing others' norms, one could manipulate whether contributions are private or public information. This would allow one to better disentangle the effects of descriptive and injunctive norms on contribution levels^46^. Third, one could vary how participants can react to each other. In our experiment, participants could react through contribution and punishment decisions. Future experiments could simplify our design by removing punishment for example, or enrich it by allowing for additional reactions through direct communication or rewards for example^47,48^.

Our study results are also relevant for research on PGGs in general. Participants entered the laboratory with their normative views already well-defined. Participants’ views mapped well along the spectrum between equal-contributions and equal-earnings, were largely stable throughout the experiment even when confronted with the conflicting behavior of others, and guided these participants’ behavior. The minds of participants entering the lab are thus not blank slates. Some scholars conjecture that participants take the social norms they adhere to in everyday life with them to the lab, and that ignoring these norms when looking at these participants’ behavior leads to substantial misinterpretations of lab experimental results^49,50^. Our study results corroborate this conjecture. The normative views participants have before they start interacting with each other strongly relate to how they behave in the subsequent interactions. Therefore, not measuring these views runs the risk of missing a large part of the variation in within-condition behavior.

One concern is that merely asking participants about norms might encourage norm compliance. However, the available evidence suggests that norm elicitation as such does not affect behavior^51^. For our study, we can directly compare the results with the results of an experiment that used the exact same game-parameter values but did not elicit norms^11^. The behavioral patterns are similar, suggesting that our norm elicitation did not substantially affect behavior (see Figure S2 in SI).

Norms are considered to be an important element in explaining human cooperative behavior across numerous disciplines^1–7^. Recently, it has been conjectured that when groups are heterogeneous rather than homogeneous, norms can also harm cooperation because they cause disagreement. We find that although there is considerable disagreement about normative views in heterogeneous groups, this disagreement does not impede cooperation. Groups cooperate despite normative disagreement, at different contribution levels depending on the average level supported by their members. Our results suggest that norms can sustain cooperation even in situations of normative disagreement.

## Methods

We conducted the computerized experiment in the Experimental Laboratory for Sociology and Economics (ELSE) at Utrecht University during October–November 2019. The experiment was programmed with z-Tree software^52^. We recruited participants amongst students at Utrecht University using the internet recruitment system ORSEE^53^. We ran 8 sessions with 24 participants each, leading to a total of 192 participants. Each session lasted about 75 min. Payment depended on behavior in the game, participants earned on average 15 euros (min = 5, max = 22). Participants were on average 24 years old, 127 (66%) were female, 62 male, and 3 other. Almost all participants were students at Utrecht University, 87 were Dutch and 105 from various other countries.

Participants were randomly placed in an individual cubicle, so they could not see, or communicate with, each other. They were informed about the experiment through written instructions (provided in the SI). There were two parts of the experiment. In the first part, participants report on their normative views and expectations, play 10 rounds of the PGG with peer punishment, and report on their normative views and expectations again. This part is designed to examine the influence of normative disagreement on public good provision. In the second part, participants are switched between groups, they play another 10 rounds of the PGG within the newly formed groups consisting of new and old members, and afterward answer questions on their normative views, expectations, meta-norms, social preferences, and background characteristics. This second part is designed to examine the influence of newcomer entry on public good provision. Participants were informed at the beginning of the experiment that there would be two parts of the experiment, but that they would only receive the information about the second part of the experiment after the first part was finished. Because we only study the influence of normative disagreement on public good provision in this paper, we only analyze the game data of the first part of the experiment. The effect of newcomer entry will be analyzed in another paper.

Each round of the PGG with peer punishment has two stages. In the first stage, each individual *i* in a group composed of 3 members receives an endowment of 20 MUs and must decide how much of this endowment to contribute to a public good, *c*_*i*_, where *c*_*i*_ є {0, 1, … , 20}. The part of the endowment that is not contributed to the public good is kept for the individual. The public good consists of the sum of the contributions made by all individuals. Each individual receives a return per contributed point (sometimes also referred to as marginal per capita return) to the public good (*m*_*i*_ < 1). The sum of these returns makes up the total multiplication factor of the public good 1.75*.* After all individuals in a group have made their contribution decision, the contributions and payoffs of each player are communicated to all and the first stage is finished.

In the second stage, each individual is given the opportunity to assign punishment points *p*_*ij*_ є {0, 1, …, 10} to each group member *j.* Each punishment point costs 1 point to the punisher, and reduces the payoff of the punished player by 3 points. The individual payoff after one round of this two-stage game is given by:

$$\pi_{i} = 20 - c_{i} + m_{i} \mathop \sum \limits_{j} c_{j} - \mathop \sum \limits_{j \ne i} p_{ij} - 3\mathop \sum \limits_{j \ne i} p_{ji}$$

Individuals do not see who punished them (to prevent confounding of normative behavior with revenge motives), and repeatedly play rounds of this two-stage game within the same group. We assign heterogeneous returns of the public good per group: one participant with a high return *m*_*i*_ = 0.75, and two participants with a lower return *m*_*i*_ = 0.50. For comparability to previous research, all other parameter values are set to follow the typical form of the PGG with peer punishment^9^.

Prior to sorting participants into groups and assigning them their returns, we present them with the game and elicit their normative view by letting the participants answer what they consider to be the appropriate contribution decisions for another hypothetical group. Each of the participants is asked to indicate the appropriate contribution for each of three group members, one with return *m*_*i*_ = 0.75, and two with *m*_*i*_ = 0.50. The participants can try out different combinations of contributions, and see how it affects the earnings of each group member (see instructions and screen shots in the SI, Figure S1). After the participants reported their personal normative view, we tell them that their group members were also asked to indicate appropriate contributions for three members in the PGG. Each of the participants is then asked to guess the answers submitted by their group members. To incentivize the guess, the participants are told that we will randomly pick one of their guesses, and give an additional payment of 100 MU (~ €1.40) when it matches the answer of at least one of the group members. Only at the end of the experiment are participants informed of whether they were correct in the guess we randomly chose. This measure is inspired by the following earlier studies^33,43,54^.

**Figure 6:**
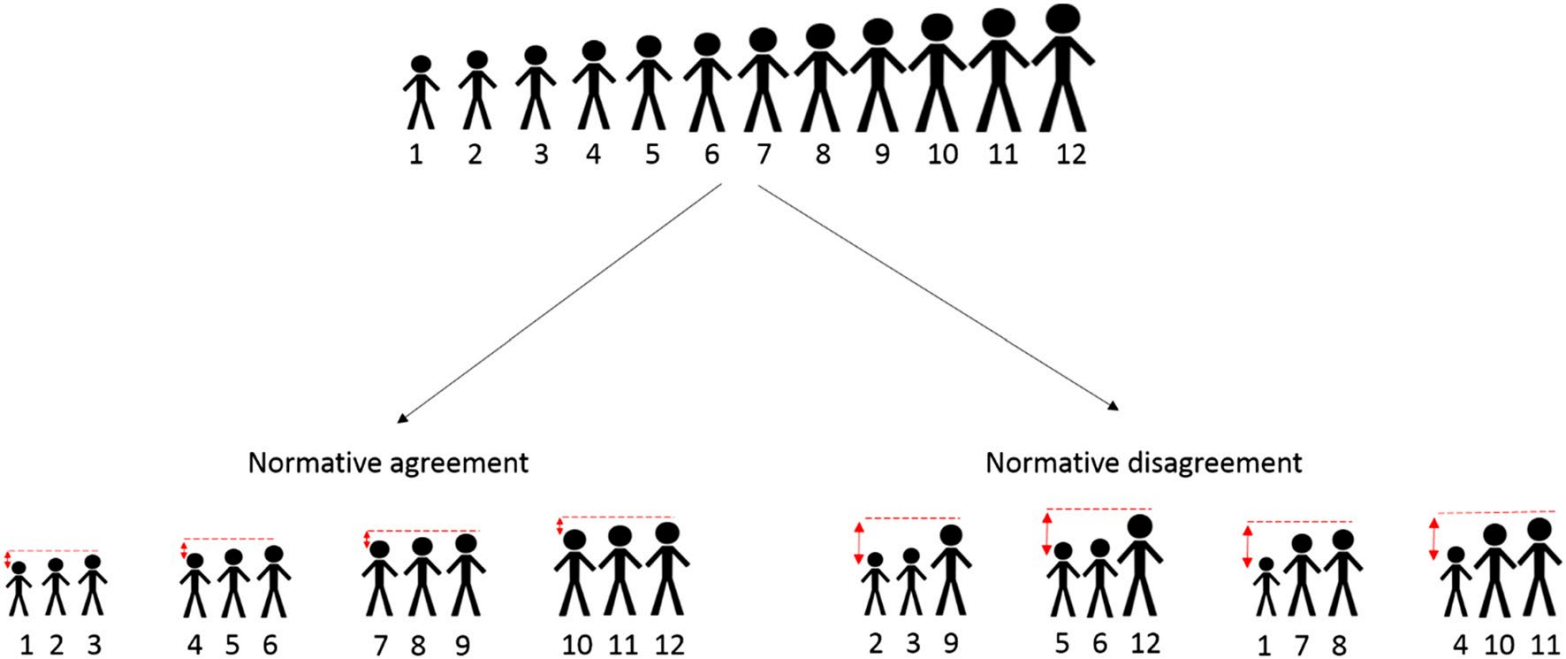
Example for method of sorting participants. At the beginning of the experiment, participants are ranked in terms of their normative views for contributions relative to returns. In the example presented here, there are 12 participants sorted into 4 groups. When sorting for normative agreement, we first form a group of the three highest-ranked participants (1–3), then of the remaining participants we again form a group of the three highest-ranked participants (4–6), and so on until all participants are grouped. Compared to the condition with normative agreement, in the condition with normative disagreement we select the highest-ranked low-return participant from the first group in the first half of the groups (ordered in terms of support for contributions relative to returns), and replace it with the lowest-ranked low-return participant from the first group in the second half of the groups, and repeat this procedure with the remaining groups. In this way, the extent of normative disagreement (in terms of rank-differences) is equal for all groups.

We sort 192 participants into 64 groups of 3 members each based on their normative views. Within each session of 24 participants, we assign each of the participants a ranking in terms of how much they support the equal-contributions rule versus the equal-earnings rule compared to the other participants in the session. The precise score used to assign ranks is: $c_{H} - \overline{c}_{L} + .02\overline{c} + .0001R$, where $c_{H}$ is the participant’s view on the appropriate contribution for the high-return member, $\overline{c}_{L}$ is the participant’s view on the appropriate contribution of the two low-return members on average, $\overline{c}$ is the mean appropriate contribution over all three members, and *R* is a random number between 0 and 1. The addition of 0.02 $\overline{c}$ makes sure that participants who assign a contribution of 20 to all members obtain slightly higher scores than participants who assign a contribution of 0 to all members. This helps to differentiate between different absolute levels of achieving the equal-contributions rule in the sorting method. The number 0.02 is chosen such that whether contributions are relative to returns or not always has dominance in the sorting mechanism over the absolute level of contributions. The addition of 0.0001*R* is to avoid tied scores. The method of sorting within the two conditions based on these scores is described in Fig. 6.

Sample size was determined based on sample sizes in comparable studies (see for example^11,12^). Indeed if the hypothesized effect would have been substantial, we would most likely have found confirmation for it. Namely, a Mann–Whitney ranksum test with individuals as unit of analyses has a high power (0.96) to detect medium-sized effects (0.5) given our sample size. With groups as unit of analyses, we have relatively low power (0.61) to detect medium-sized effects (0.5), but high power (0.92) to detect large effects (0.8). These estimates are conservative as they are based on one observation per individual/group while we have ten (correlated) observations per individual/group (one observation per round). The effects of normative disagreement that we find do not reach statistical significance either at the individual or group level and are small in terms of effect size.

We decided not to inform participants about the method of group formation for three main reasons. First, we modelled our design on prior studies that gave rise to the conjecture that normative disagreements harm cooperation^11^ (see for example the comparison in the supplementary material, Figure S2). The main difference we introduced was our addition of the norm elicitation and the associated sorting based on this norm elicitation, both of which do not directly affect the participants' beliefs. Had we also told participants about the method of group formation, we would likely have altered the participants’ beliefs on how cooperative their group members are, which makes comparison with other studies more difficult. Second, by telling participants that their normative views will be used for group formation, we might create experimenter demand effects on the importance of these views for behavior. That is, it might lead the participants to believe that the experimenter judges these normative views to be important for behavior in the game. Participants who want to comply with the experimenter’s belief may as a result act more in line with their normative views once they know about the sorting procedure. Third, revealing that the normative views will be used for group formation may provide participants with an incentive to misrepresent their normative views. For example, in the normative agreement condition, if participants know that they will be grouped according to their normative views with similar others, they might report their normative views to be more prosocial than they actually are, so as to be grouped with others that hold prosocial views. Note that although we did not reveal how grouping was done, we did not offer untruthful information to the participants about the group formation, e.g., we did not say the formation was random. Instead, we told the participants when the formation had happened (directly after the norm elicitation), but not how it had happened.

After reading the instructions, participants were given six questions to test their understanding of the game. Upon completion, they were shown which questions they had answered correctly and incorrectly. At the first try, 158 participants answered 5–6 questions correctly, 26 participants answered 3–4 questions correctly, and 8 participants answered less than 3 questions correctly. Participants had to redo all questions that they answered incorrectly until all answers were correct. At the end of the experiment, participants were asked to rate whether their understanding of the experiment was (1) bad, (2) not bad, not good, or (3) good. 169 participants reported a good understanding, 22 reported not bad, not good, and 1 reported a bad understanding. These figures give us confidence that the experiment was adequately understood. Throughout the experiment, participants had the opportunity to ask questions to the lab official, less than a handful did so. The experimental data is openly available at: 10.24416/UU01-87KATL. The experiment was preregistered before data collection at Open Science Framework, osf.io/gy8st. Informed consent was obtained from all participants and the experimental procedures were approved by the Ethics Committee of the Faculty of Social and Behavioral Sciences of Utrecht University. All research was in line with relevant regulations.

## Supplementary information

Supplementary Information.
