# Supplementary material for: Heterogeneous groups cooperate in public good problems despite normative disagreements about individual contribution levels
Source: Sci Rep. 2020 Oct 7;10:16702. doi: 10.1038/s41598-020-73314-7 (PMC7542426; doi:10.1038/s41598-020-73314-7)
Supplement: Supplementary file 1 — Supplementary Information. [file 41598_2020_73314_MOESM1_ESM.pdf]

Supplementary information for article:

Heterogeneous groups cooperate in public good problems despite normative disagreements about individual contribution levels

Kasper Otten[1,*], Vincent Buskens[1], Wojtek Przepiorka[1], Naomi Ellemers[2]

[1] Utrecht University, Department of Sociology
[2] Utrecht University, Department of Psychology

[*] Corresponding author: Padualaan 14, 3584 CH Utrecht, the Netherlands; k.d.otten@uu.nl; +31 30 253 8813

Figure S1a-b. Screenshots of experimental normative view measurement

(a) before calculating the payoff consequences of one's normative view

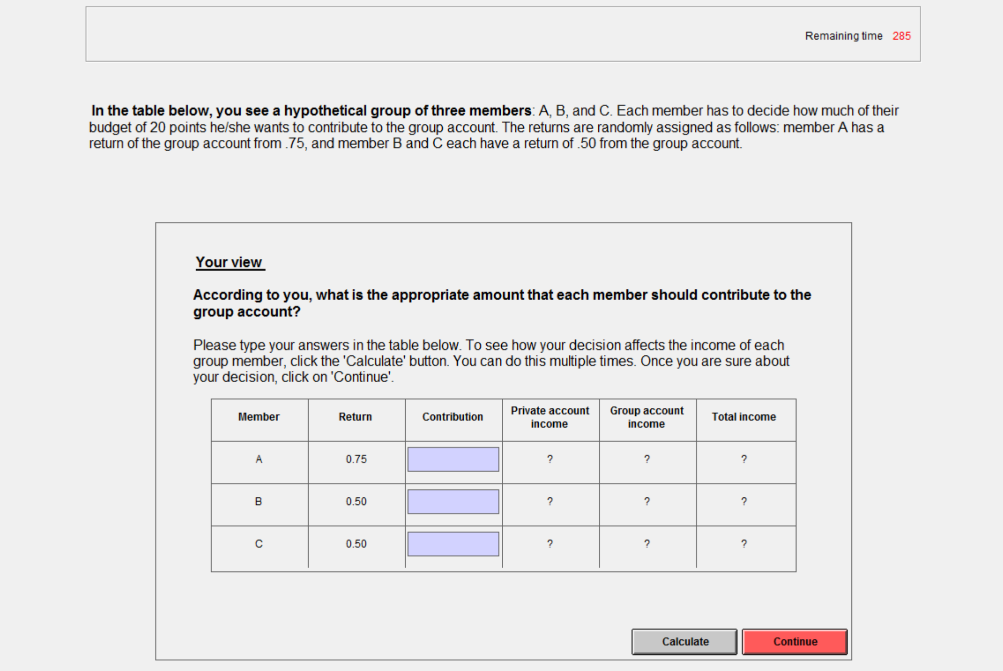

Remaining time 285

In the table below, you see a hypothetical group of three members: A, B, and C. Each member has to decide how much of their budget of 20 points he/she wants to contribute to the group account. The returns are randomly assigned as follows: member A has a return of the group account from .75, and member B and C each have a return of .50 from the group account.

Your view

According to you, what is the appropriate amount that each member should contribute to the group account?

Please type your answers in the table below. To see how your decision affects the income of each group member, click the 'Calculate' button. You can do this multiple times. Once you are sure about your decision, click on 'Continue'.

Member	Return	Contribution	Private account income	Group account income	Total income
A	0.75		?	?	?
B	0.50		?	?	?
C	0.50		?	?	?

Calculate Continue

(b) after calculating the payoff consequences of one's normative view

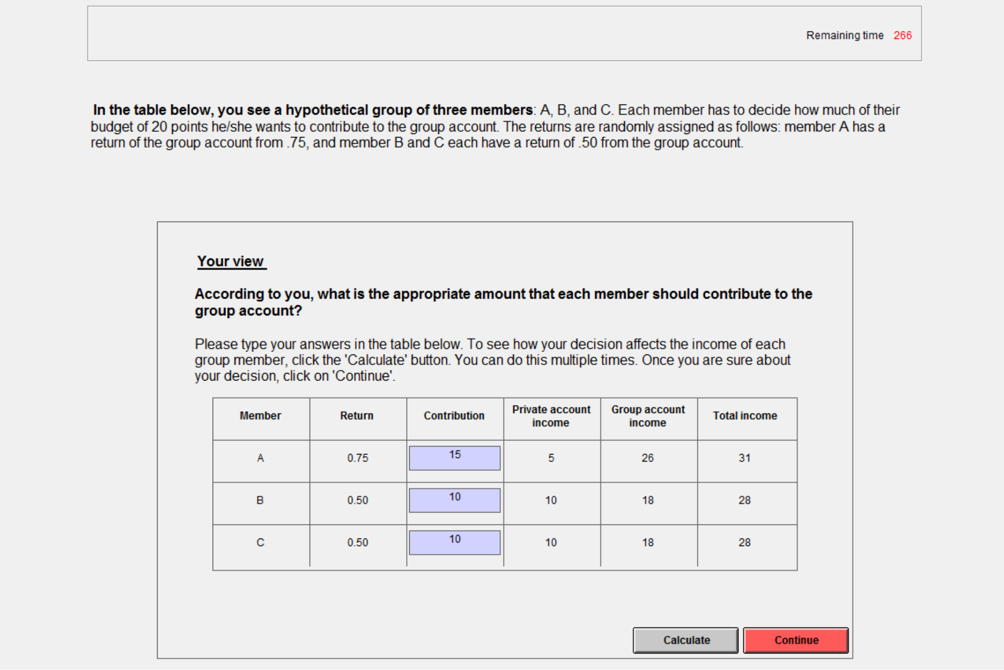

Remaining time 266

In the table below, you see a hypothetical group of three members: A, B, and C. Each member has to decide how much of their budget of 20 points he/she wants to contribute to the group account. The returns are randomly assigned as follows: member A has a return of the group account from .75, and member B and C each have a return of .50 from the group account.

Your view

According to you, what is the appropriate amount that each member should contribute to the group account?

Please type your answers in the table below. To see how your decision affects the income of each group member, click the 'Calculate' button. You can do this multiple times. Once you are sure about your decision, click on 'Continue'.

Member	Return	Contribution	Private account income	Group account income	Total income
A	0.75	15	5	26	31
B	0.50	10	10	18	28
C	0.50	10	10	18	28

Calculate Continue

Table S1. Changes in normative views for minority and majority participants

	Position of participant		Mann-Whitney test of difference by return-type	
	minority	majority	*z*-statistic	*p*-value
Absolute change between rounds 1 - 10				
normative view on high-return members	2.38	2.70	0.94	.35
	(4.35)	(3.56)		
normative view on low-return members	2.50	2.44	-0.23	.82
	(3.57)	(3.42)		
Relative change between rounds 1 - 10				
normative view on high-return members	2.38	1.39	-1.12	.26
	(4.35)	(4.26)		
normative view on low-return members	0.94	1.65	0.78	.44
	(4.27)	(3.87)		

Note: estimates are from condition disagreement because only when there is disagreement can we say one participant holds a minority view and the other two hold a majority view. For the absolute change, both positive and negative changes are regarded as positive levels of change. For the relative change, the negative changes are subtracted from the positive ones. Positive changes indicate that the participants' normative views prescribe higher contributions in round 10 than in round 1. Standard deviations are in parentheses.

Table S2. Mann-Whitney test for contributions and punishments by condition

Contributions - individual level	
z-statistic	-2.235
Contributions - group level	
z-statistic	-0.994
Punishment - individual level	
z-statistic	2.049
Punishment - group level	
z-statistic	1.372

Note: We test whether the contribution and punishment decisions over rounds 1-10 differ between conditions with non-parametric Mann-Whitney ranksum tests, both when using individual decisions and group-mean decisions. We find no significant differences according to conventional standards: * $p < 0.05$, ** $p < 0.01$, *** $p < 0.001$ (Bonferroni-adjusted $p/4$, two-tailed tests)

Figure S2. Behavioral trends in Otten et al. (2020) and Reuben and Riedl (2013)

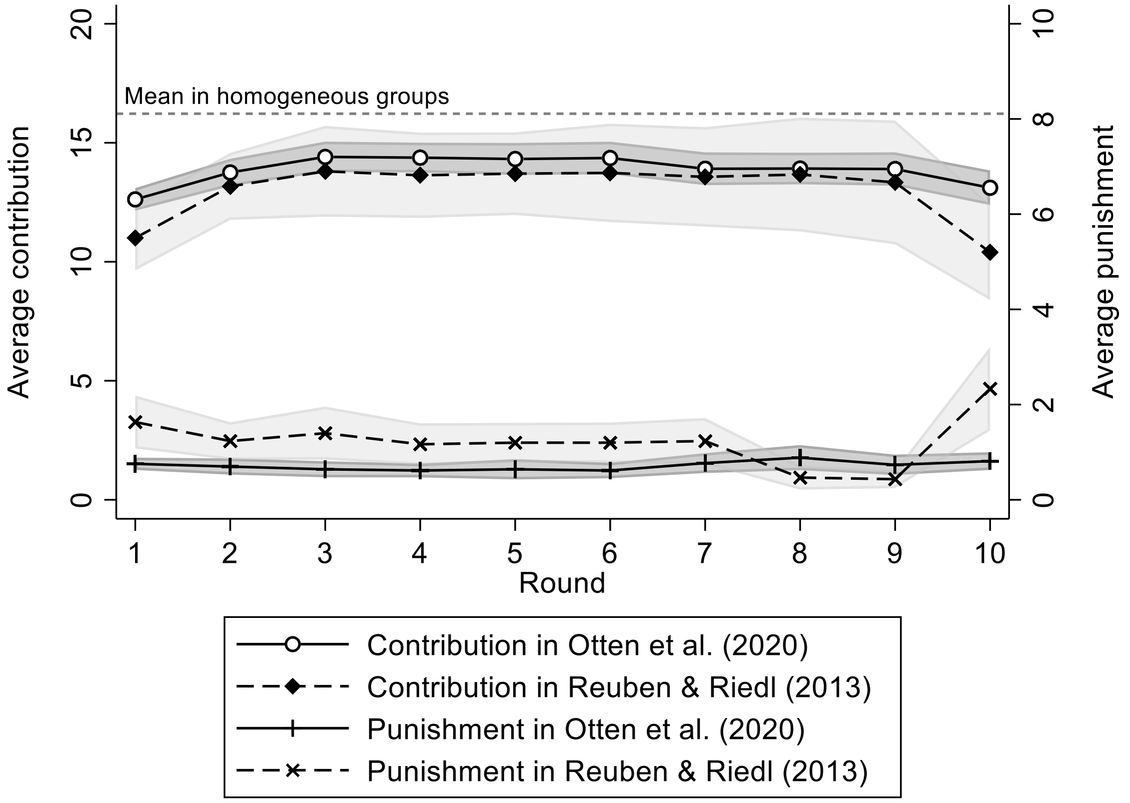

Note: N = 1920 (192 participants x 10 rounds) for Otten et al. (2020) and N = 300 (30 participants x 10 rounds) for Reuben and Riedl (2013). The main experimental parameter values were exactly the same in both studies, but Otten et al. (2020) measured norms before play whereas Reuben and Riedl (2013) did not. That the behavioral patterns are largely similar across the two studies suggests that the norm measurement did not affect behavior. 95% confidence intervals are added for all outcomes on the group-level with grey area shading. A dashed horizontal line is added displaying the average contribution level found in the homogeneous groups with peer punishment in Reuben and Riedl (2013). The contribution levels in the heterogeneous conditions are below that of the homogeneous condition. Otten et al. (2020) refers to the current manuscript, Reuben and Riedl (2013) refers to: Reuben, E. & Riedl, A. Enforcement of contribution norms in public good games with heterogeneous populations. *Games Econ. Behav.* **77**, 122–137 (2013).

Figure S3a-b. Average contribution and punishment per round and condition and return-type

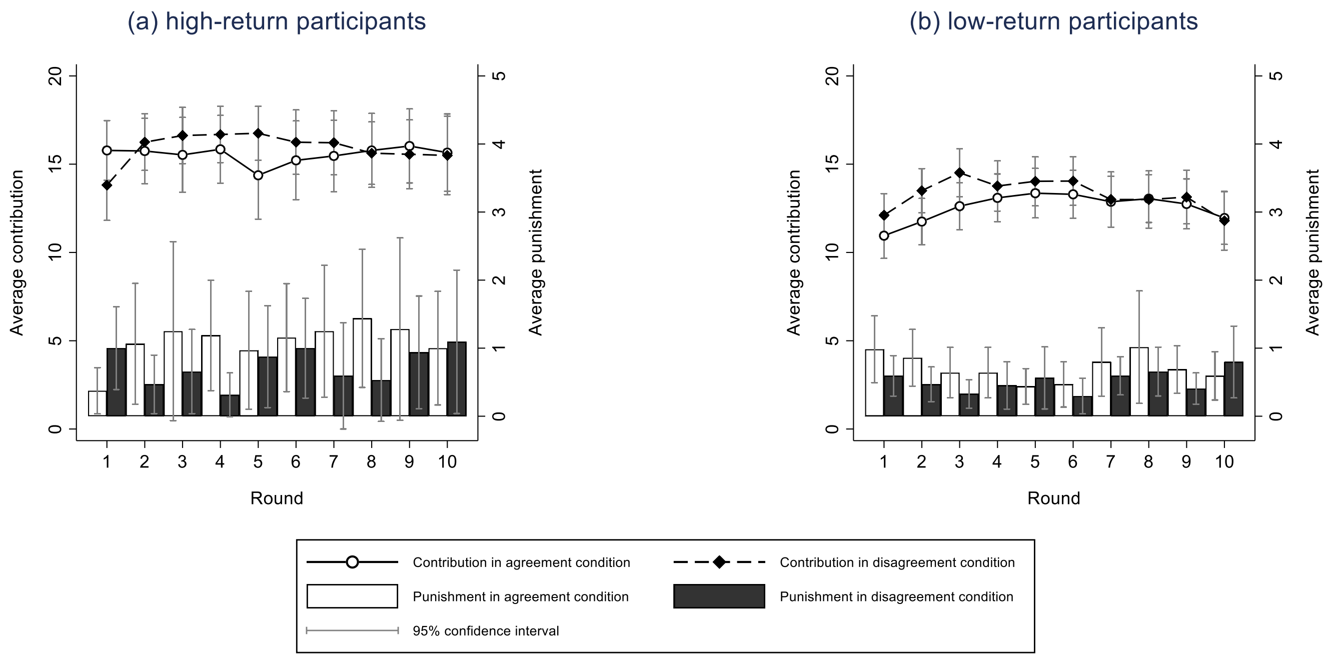

Figure S4a-c. Contributions by normative views of high-return participants

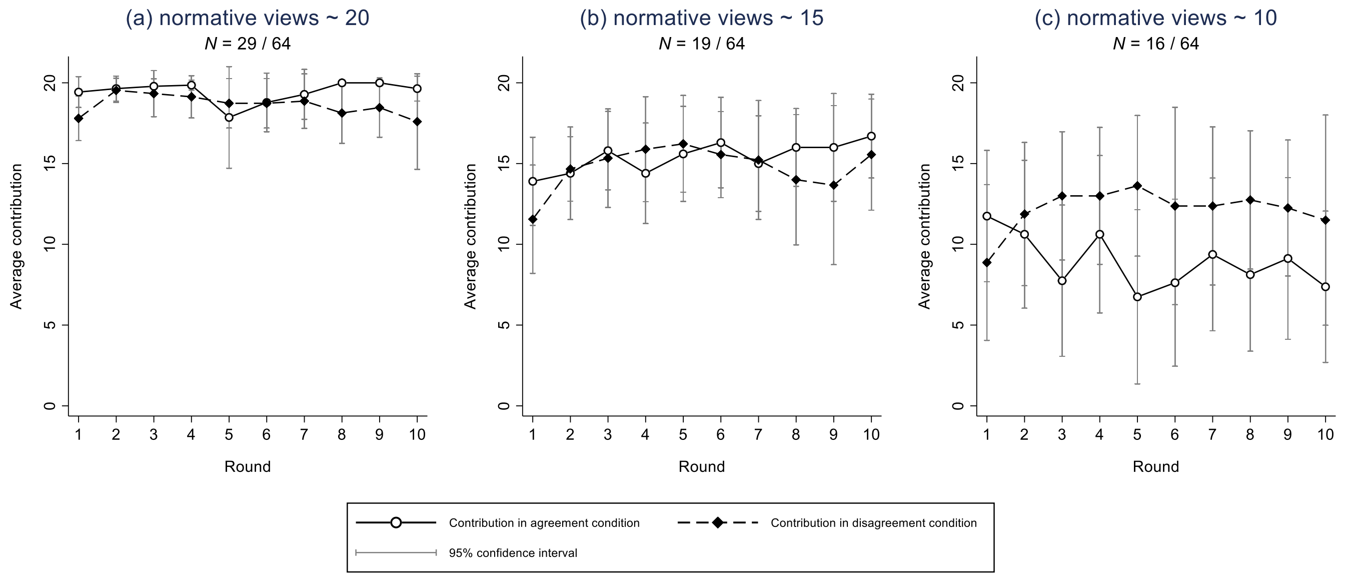

Note: We examine contribution levels for high-return participants according to their first-measured normative views of what high-return participants should contribute, i.e., what they think what would be appropriate contributions for themselves. We distinguish three levels: (1) normative views that prescribe contributions of ~ 20 (≥ 17.5), (2) normative views that prescribe contributions of ~ 15 (< 17.5 & ≥ 12.5), and (3) normative views that prescribe contributions of ~ 10 (< 12.5 & ≥ 7.5). 1 high-return participant reported that a contribution of 5 is appropriate for high-return participants. We did not think it useful to create a separate category for 1 participant, and therefore included the participant in the category with normative views ~ 10.

Figure S5a-d. Contributions by normative views of low-return participants

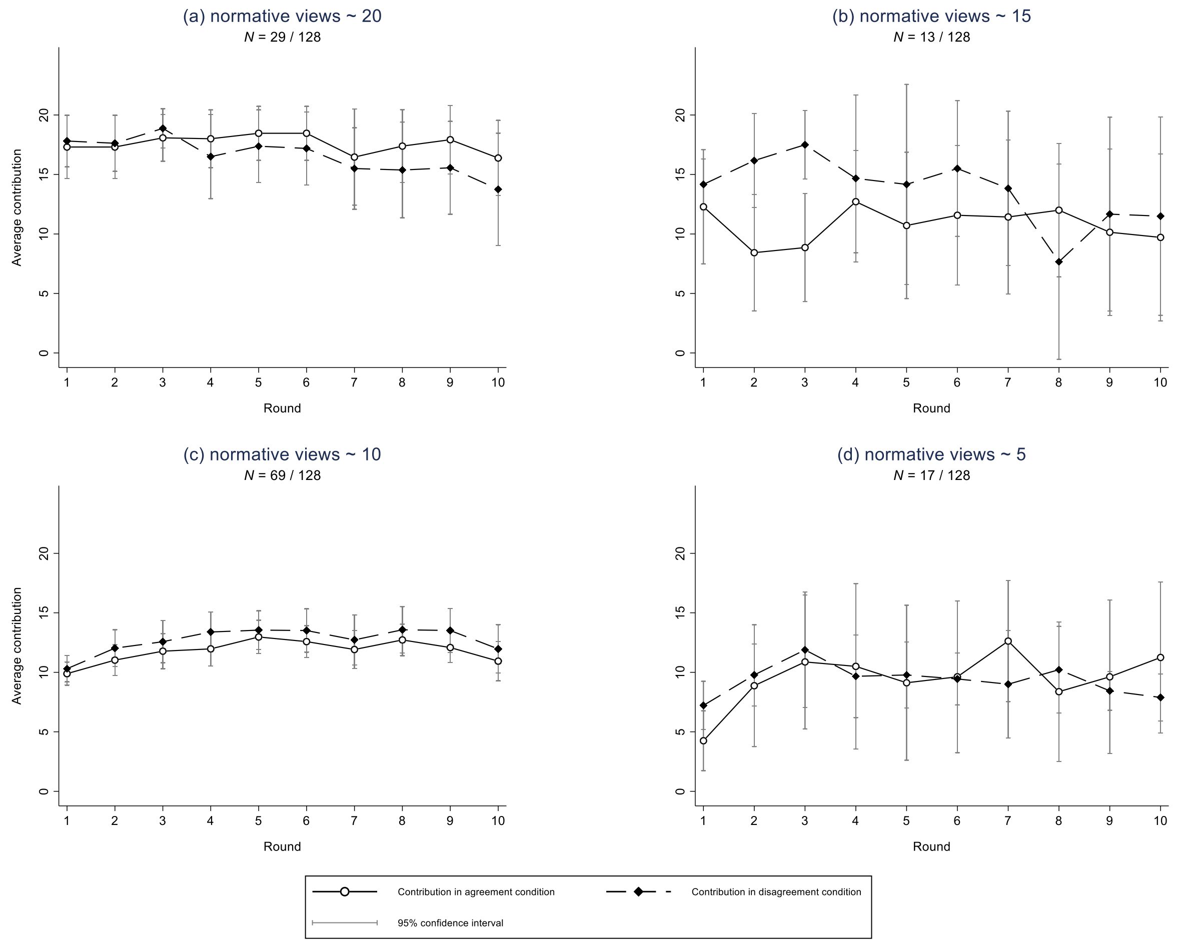

Note: We examine contribution levels for low-return participants according to their first-measured normative views of what low-return participants should contribute, i.e., what they think what would be appropriate contributions for themselves. We distinguish four levels: (1) normative views that prescribe contributions of ~ 20 (≥ 17.5), (2) normative views that prescribe contributions of ~ 15 (< 17.5 & ≥ 12.5), (3) normative views that prescribe contributions of ~ 10 (< 12.5 & ≥ 7.5), and (4) normative views that prescribe contributions of ~ 5 (< 7.5).

Exploratory analyses

Because virtually all variation in contribution levels is within conditions, we turn to exploratory analyses to examine what might explain this variation. In the explanatory analyses, we conceptualized normative disagreement based on normative views about equal-contributions and equal-earnings as is common in related studies. We now take three main alternative approaches to assess how normative disagreement can affect cooperation in the PGG. First, some perspectives suggest that rather than to personal normative views (i.e., what participants themselves deem appropriate), participants respond to normative expectations (i.e., what participants think their group members deem appropriate). The corresponding prediction would be that disagreement in normative expectations, rather than personal views, will negatively affect the level of public good provision. Second, next to disagreement on how much high-return members should contribute relative to low-return members (equal-earnings vs equal-contributions), we examine disagreement on absolute contribution norms. That is, there are multiple absolute levels of contributions that may satisfy the rules of equal-earnings or equal-contributions, and people may disagree about the appropriate absolute level as well. Third, for normative disagreement to have the potential to reduce public good provision, norm conformity must be conditional on the conformity of others. An alternative perspective is that people conform to their normative views unconditionally, in which case the group-mean normative view, rather than disagreement on these views, predicts public good provision.

Altogether, we thus examine the dimensions of (1) normative views vs normative expectations, (2) absolute versus relative contribution rules, and (3) group-mean norms versus group-disagreement on norms. The combination of all three dimensions gives us 8 (2x2x2) potential ways in which norms may influence public good provision. We conduct a random-effects Tobit regression with each of the 8 norm-conceptualizations as independent variables and the group-mean contribution per round as the dependent variable. All variables are standardized, to allow for comparisons of their effect sizes. Figure S6 presents the results of the analysis (see also Table S3).

Figure S6. Effects of normative views and expectations on public good provision

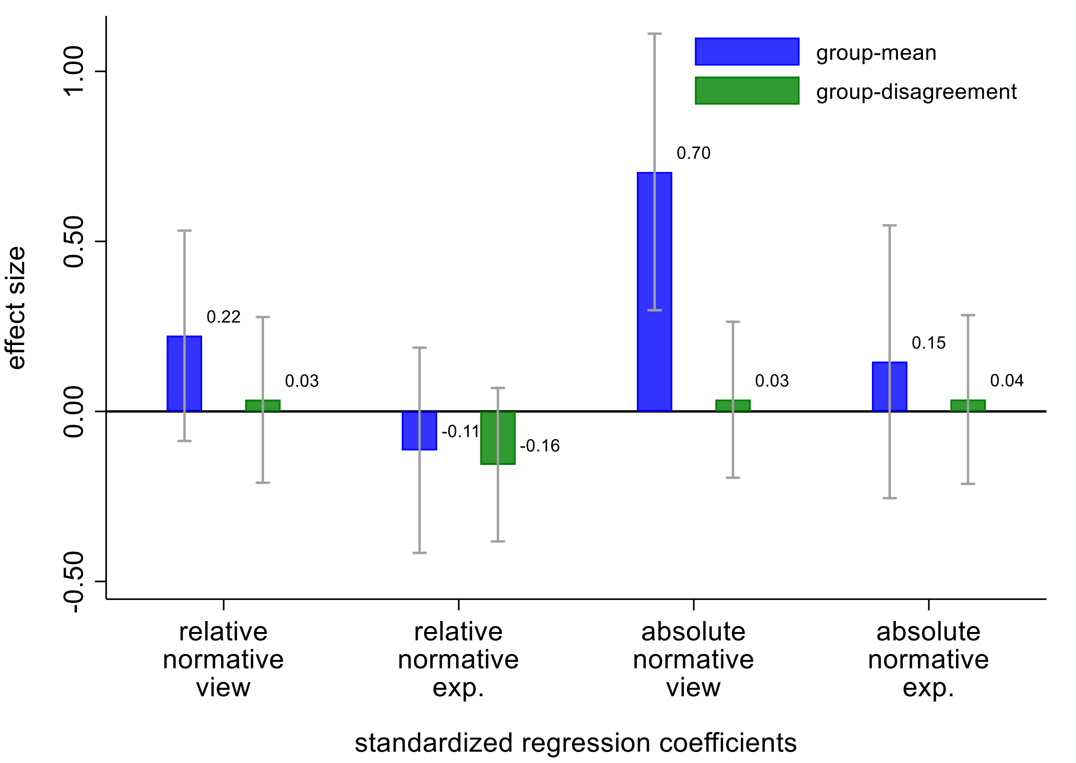

Note: Per predictor, the mean group-score is the average of all 3 participants, and the disagreement score is the group's maximum score subtracted by the group's minimum. Relative normative view (and expectation) refers to the difference in the appropriate contribution between the high- and low-return members. Absolute normative view (and exp.) refers to the appropriate absolute level of contributions averaged over the low- and high-return members. The participants' normative views are what they themselves deem appropriate, the participants' normative expectations are what they expect their group members to deem appropriate. The 95% confidence intervals are included and Bonferroni adjusted for multiple testing. Full model specification is shown in Table S3, column 5.

Figure S6 corroborates the conclusion that normative disagreement between the relative contribution rules of equal-contributions and equal-earnings does not affect public good provision: group-disagreement on relative normative views does not significantly affect the group-mean contribution. Because there are multiple absolute levels of contributions that may satisfy the rules of equal-earnings or equal-contributions, people may disagree about the appropriate absolute level as well. In Figure 2a of the main-text, we indeed see that among supporters of each rule, there is variation in the absolute contribution levels considered appropriate. However, in Figure S6 we see that such group-disagreement on the absolute normative views does not affect the contribution level. Similarly, whether disagreement is in terms of normative views or normative expectations also does not seem to matter: both group-disagreements on views and expectations are unrelated to public good provision. Thus, regardless of how we conceptualize normative disagreement, it does not seem to significantly affect public good provision.

Table S3. Random-effects Tobit Regression of Mean Public Good Provision

	(1) relative normative view	(2) relative normative exp.	(3) absolute normative view	(4) absolute normative exp.	(5) all norms w/o controls	(6) all norms with controls
[mean] relative normative view	-.081				.222	.183
	(.138)				(.113)	(.109)
[dis] relative normative view	-.145				.034	.044
	(.138)				(.089)	(.087)
[mean] relative normative exp.		-.190			-.114	-.115
		(.127)			(.110)	(.105)
[dis] relative normative exp.		-.194			-.157	-.231*
		(.127)			(.083)	(.080)
[mean] absolute normative view			.809***		.704***	.666***
			(.085)		(.149)	(.148)
[dis] absolute normative view			-.014		.034	-.025
			(.084)		(.084)	(.083)
[mean] absolute normative exp.				.732***	.146	.130
				(.096)	(.147)	(.139)
[dis] absolute normative exp.				-.111	.035	-.024
				(.094)	(.091)	(.089)
[mean] social value orientation						-.137
						(.085)
[dis] social value orientation						-.132
						(.088)
[mean] age						.094
						(.076)
[mean] political orientation						-.131
						(.078)
proportion of males						.170
						(.080)
round	.013	.013	.013	.013	.013	.013
	(.008)	(.008)	(.008)	(.008)	(.008)	(.008)
constant	.033	.034	.035	.037	.035	.034
	(.137)	(.133)	(.094)	(.103)	(.089)	(.083)
sigma_u	1.017***	.987***	.638***	.722***	.586***	.528***
	(.095)	(.092)	(.062)	(.069)	(.058)	(.053)
sigma_e	.567***	.567***	.567***	.567***	.567***	.567***
	(.019)	(.019)	(.019)	(.019)	(.019)	(.019)
N observations	640	640	640	640	640	640
N groups	64	64	64	64	64	64
rho	.763	.752	.559	.619	.517	.464

Note: We conduct random-effects Tobit regressions with each of the norm-conceptualizations as independent variables (see main text) and the group-mean contribution per round as the dependent variable. For each variable, [mean] refers to the group-mean score and [dis] refers to the group-disagreement score. In all models, 1 observation is left-censored and 123 observations are right-censored. In columns 1-4, the group-mean and group-disagreement on the different norm-conceptualizations are tested in separate models. In column 5, all norm-conceptualizations are included within a single model, and in column 6 control variables are added. We control for the group composition on several variables measured at the end of the experiment: social value orientation, sex, age, and political orientation (self-reported from 1 = very left to 10 = very right). All variables are standardized (except round), to allow for comparisons of their effect sizes. * $p < 0.05$, ** $p < 0.01$, *** $p < 0.001$ (Bonferroni-adjusted $p/8$, two-tailed tests). Standard errors in parentheses. As can be seen, the only variable strongly related to the group-mean contribution is the group-mean absolute normative view. The only exception is the group-mean absolute normative expectation in model 4, but that is because normative expectations are highly correlated with normative views as reported in the main text. The effect of group-mean normative expectations disappears when controlling for group-mean normative views.

Table S4. OLS Regression of Mean Public Good Provision

	(1) relative normative view	(2) relative normative exp.	(3) absolute normative view	(4) absolute normative exp.	(5) all norms w/o controls	(6) all norms with controls
[mean] relative normative view	-.018				.230	.205
	(.117)				(.090)	(.098)
[dis] relative normative view	-.106				.048	.064
	(.102)				(.062)	(.059)
[mean] relative normative exp.		-.116			-.073	-.080
		(.105)			(.105)	(.096)
[dis] relative normative exp.		-.181			-.151	-.220
		(.122)			(.084)	(.089)
[mean] absolute normative view			.660***		.589***	.556***
			(.067)		(.125)	(.134)
[dis] absolute normative view			-.007		.042	-.010
			(.072)		(.068)	(.063)
[mean] absolute normative exp.				.594***	.123	.110
				(.076)	(.125)	(.119)
[dis] absolute normative exp.				-.092	.038	-.017
				(.076)	(.081)	(.080)
[mean] social value orientation						-.127
						(.085)
[dis] social value orientation						-.129
						(.078)
[mean] age						.078
						(.052)
[mean] political orientation						-.097
						(.074)
proportion of males						.154
						(.077)
round	.002	.002	.002	.002	.002	.002
	(.012)	(.012)	(.012)	(.012)	(.013)	(.013)
constant	-.012	-.012	-.012	-.012	-.012	-.012
	(.112)	(.114)	(.067)	(.072)	(.068)	(.073)
N observations	640	640	640	640	640	640
N groups	64	64	64	64	64	64
R2	.010	.051	.437	.362	.495	.543

Note: We conduct ordinary least squares (OLS) regression with each of the norm-conceptualizations as independent variables (see main text) and the group-mean contribution per round as the dependent variable. For each variable, [mean] refers to the group-mean score and [dis] refers to the group-disagreement score. We account for repeated measures within groups by estimating cluster-robust standard errors. In columns 1-4, the group-mean and group-disagreement on the different norm-conceptualizations are tested in separate models. In column 5, all norm-conceptualizations are included within a single model, and in column 6 control variables are added. We control for the group composition on several variables measured at the end of the experiment: social value orientation, sex, age, and political orientation (self-reported from 1 = very left to 10 = very right). All variables are standardized (except round), to allow for comparisons of their effect sizes. * $p < 0.05$, ** $p < 0.01$, *** $p < 0.001$ (Bonferroni-adjusted $p/8$, two-tailed tests). Standard errors in parentheses.

Table S5. Population-Averaged Regression of Mean Public Good Provision

	(1) relative normative view	(2) relative normative exp.	(3) absolute normative view	(4) absolute normative exp.	(5) all norms w/o controls	(6) all norms with controls
[mean] relative normative view	-.047				.191	.161
	(.109)				(.089)	(.086)
[dis] relative normative view	-.110				.034	.044
	(.109)				(.070)	(.069)
[mean] relative normative exp.		-.112			-.049	-.052
		(.100)			(.086)	(.083)
[dis] relative normative exp.		-.157			-.137	$-.202^{*}$
		(.100)			(.065)	(.063)
[mean] absolute normative view			$.640^{***}$		$.568^{***}$	$.537^{***}$
			(.067)		(.116)	(.117)
[dis] absolute normative view			.007		.047	-.007
			(.067)		(.066)	(.065)
[mean] absolute normative exp.				$.573^{***}$	.124	.114
				(.073)	(.114)	(.109)
[dis] absolute normative exp.				-.077	.045	-.011
				(.073)	(.070)	(.069)
[mean] social value orientation						-.119
						(.067)
[dis] social value orientation						-.107
						(.070)
[mean] age						.096
						(.060)
[mean] political orientation						-.109
						(.061)
proportion of males						.137
						(.063)
round	.011	.011	.010	.010	.009	.009
	(.016)	(.016)	(.014)	(.014)	(.013)	(.013)
constant	-.198	-.194	-.150	-.161	-.138	-.129
	(.134)	(.131)	(.101)	(.108)	(.095)	(.090)
N observations	640	640	640	640	640	640
N groups	64	64	64	64	64	64

Note: We conduct population-averaged regressions with each of the norm-conceptualizations as independent variables (see main text) and the group-mean contribution per round as the dependent variable. For each variable, [mean] refers to the group-mean score and [dis] refers to the group-disagreement score. In columns 1-4, the group-mean and group-disagreement on the different norm-conceptualizations are tested in separate models. In column 5, all norm-conceptualizations are included within a single model, and in column 6 control variables are added. We control for the group composition on several variables measured at the end of the experiment: social value orientation, sex, age, and political orientation (self-reported from 1 = very left to 10 = very right). All variables are standardized (except round), to allow for comparisons of their effect sizes. The within-subject working correlation matrix is autoregressive of order 1. * $p < 0.05$, ** $p < 0.01$, *** $p < 0.001$ (Bonferroni-adjusted $p/8$, two-tailed tests). Standard errors in parentheses.

Figure S7. Normative views on punishment of free-riders (a), adherents to equal-earnings norm (b), and unconditional prosocials (c)

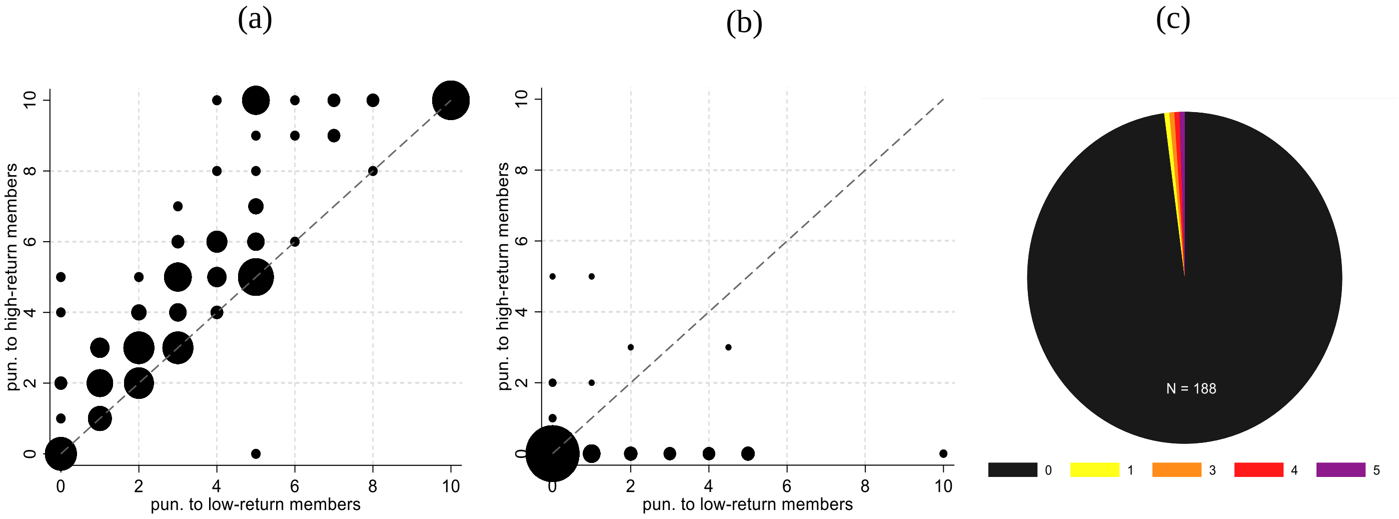

Note: Participants were asked to report on their normative views and expectations of punishment in two hypothetical scenarios after completion of the experiment. In both scenarios, one unit of punishment costs the punisher one MU and reduces the punished member's income by 3 MUs, as in the actual experiment. In the first scenario, 1 low-return member contributed all 20 points and the two other members (one low- and one high-return member) contributed 0. These last two members are the free-riders, and the normative views on how much they should be punished are plotted in panel (a). Appropriate number of punishment points are plotted on the *x*-axis for the low-return members and on the *y*-axis for the high-return member. The member who contributed 20 in this scenario is the unconditional prosocial. The distribution of normative views on the appropriate punishment for this member are presented via a pie chart in panel (c). In the second hypothetical scenario, the high-return member contributed 20 points and the two low-return members each contributed 10 points, which leads to equal earnings for all. The normative views on the appropriate punishment for these low- and high-return members who adhere to the equal-earnings norm are presented in panel (b). Appropriate number of punishment points are plotted on the *x*-axis for the low-return members and on the *y*-axis for the high-return member. Because normative views and expectations on punishment are highly correlated, we only present the normative views in all three panels. We see that, while most participants agree that some level of punishment is appropriate for free-riders (although there is large variation in how much is considered appropriate), most participants agree that no punishment is appropriate for adherents to the equal-earnings norm. Yet, when asking about contribution norms, only a minority of participants reports that equal-earnings is the appropriate norm (the majority balances equal-contributions and equal-earnings, see Figure 2a in main text). This suggests that participants are tolerant of behaviors that conform to other normative views, as long as these normative views are to some extent prosocial. A norm of free-riding is largely condemned, but the equal-earnings norm is tolerated even by those who do not subscribe to the equal-earnings norm themselves. Punishment of members who contribute fully while others are free-riding is virtually never supported.

# - Instructions -

Welcome

Welcome to this experiment and thank you for coming. Please read the following instructions carefully. These instructions are the same for all participants. The instructions state everything you need to know in order to participate in the experiment. If you have any questions, please raise your hand. One of the experimenters will approach you and answer your question.

The experiment is about group decision making. You can earn money by means of earning points during the experiment. The number of points that you earn depends on your own choices, the choices of other participants in your group, and chance. At the end of the experiment, the total number of points that you earned will be exchanged at a rate of:

70 points = 1 Euro

The money you earn will be rounded up to whole euros and paid out in cash at the end of the experiment. There is a minimum payment of 5 euros, and a maximum payment of 23 euros. Other participants will not see how much you have earned. During the experiment you are not allowed to communicate with other participants. Please turn off your mobile phone. You may only use functions on the computer screen that are necessary to carry out the experiment.

First, we introduce the decision situation in which you will interact. You will learn about the procedure of the experiment later. **A decision situation consists of 2 stages: a contribution stage followed by a review stage.** In the contribution stage, you decide how many points you contribute to a group account. In the review stage, you learn how much the other members of your group contributed to the group account. We will first explain the contribution stage.

Contribution stage

You are a member of a group of **3 participants**. You and the two other members of your group are **each given 20 points**. Each of you can **choose how many points to keep for yourself in a private account and how many points to contribute to a group account**.

<u>**Your points from the private account**</u>
You will earn 1 point for each point you keep in your private account.
For example, if you keep all 20 points into your private account (and therefore do not contribute to the group account), your income will amount to exactly 20 points out of your private account. If you keep 6 points into your private account, your income from this account will be 6 points. **No one except you earns something from your private account.**

<u>**Your income from the group account**</u>
Each group member will profit from points you contribute to the group account. You will also profit from the other group members' contributions. Just like in real life, some persons profit more from contributions to the group account than others.

For each point contributed to the group account (by you and the other members):
1 member earns 0.75 points and 2 members earn 0.50 points each.

Whether you are a member with a return of 0.50 or 0.75 from the group account will be randomly determined at the start of the experiment, **and will stay the same for the entire duration of the experiment**.

For example, if the 3 members combined contribute in **total 40 points to the group account**,

1 member receives: **0.75 times 40 = 30 points** from the group account,
2 members each receive: **0.50 times 40 = 20 points** from the group account.

<u>Your total income from the private account and group account</u>

Each member can choose any number of points to contribute to the group account, from 0 to 20 points. Every point a member does not contribute to the group account will automatically remain in his/her private account. **Each member's total income from the contribution stage is the combined income from his/her private account and the group account.**

Table 1 gives an arbitrary example of how each member's income from the private account, group account, and the total income are calculated when the total contributions to the group account are 40 (15+15+10).

Table 1 – example

Member	Return	Contribution	Private account income	Group account income	Total income
A	0.75	15	5	30	35
B	0.50	15	5	20	25
C	0.50	10	10	20	30

Review stage

Each contribution stage is followed by a review stage. In the review stage, everyone in the group will see how much each of the other group members contributed to the group account as well as their income from the contribution stage. Then, all group members have a chance to **decrease** the income of each other group member. You can decide if you want to spend points to decrease the income of the other two group members, for example because you disagree with how much they contributed or earned.

If you want to decrease another member's income you do that by assigning deduction points. **Every deduction point assigned to another group member reduces his/her income by 3 points, and your own income by 1 point**. Similarly, every deduction point that one of your group members assigns to you decreases your income by 3 points and costs the group member 1 point. Note that this might imply that you or other participants lose income in a particular round. If you do not want to decrease the income of a group member, you must assign him/her

0 deduction points. Every participant can assign up to a maximum of 10 deduction points to each group member, regardless of the income from the contribution stage.
For example, if you assign 2 deduction points to a group member this costs you 2 points and reduces the group member's income by 6 points (2 times 3). Another example: if one of your group members assigns 3 deduction points to you, this reduces the group member's income by 3 points and your income by 9 points (3 times 3).

After everyone has made a decision, you will see how many deduction points were assigned to you by the other group members and also what your total income for the round is. You will not see which individual participant assigned deduction points to you, you can only see the total number of deduction points assigned to you and how that affected your income. Similarly, if you assigned deduction points to one or more of your group members, they will not see that you are the one who assigned the points.

Overview of the Session

The experiment consists of **2 parts**, and in total lasts about **1 hour and 45 minutes**.

In the 1st part you will play 10 rounds of the decision situation (10 contribution and 10 review stages).

Before you play these 10 rounds, we will first ask you to answer some questions about the decision situation. These questions concern:

- your understanding of the decision situation,
- your view on the appropriate amount that each group member should contribute to the group account,
- your guess of what the other participants think are appropriate contributions.

Some questions appear multiple times throughout the experiment. You do not have to be consistent with your answers to these questions. Your answers may or may not have changed during the experiment. Similarly, what you view as appropriate contributions may or may not be the same as what the other participants think are appropriate contributions.

After this 1st part in which you answer questions about the decision situation and play 10 rounds of it, **you will receive new instructions on your computer screen for the 2nd part of the experiment.** The 2nd part of the experiment is of similar length to the 1st part.

Because you play together with other persons, you will sometimes have to wait until the other persons have made their decision. These waiting times are incorporated in the total expected duration of 1 hour and 45 minutes for the experiment.
